# MOS11: A New Component in the mRNA Export Pathway

**DOI:** 10.1371/journal.pgen.1001250

**Published:** 2010-12-23

**Authors:** Hugo Germain, Na Qu, Yu Ti Cheng, EunKyoung Lee, Yan Huang, Oliver Xiaoou Dong, Patrick Gannon, Shuai Huang, Pingtao Ding, Yingzhong Li, Fred Sack, Yuelin Zhang, Xin Li

**Affiliations:** 1 Michael Smith Laboratories, University of British Columbia, Vancouver, Canada; 2 Natural Resources Canada, Canadian Forest Service, Laurentian Forestry Centre, Stn. Sainte-Foy, Canada; 3 National Institute of Biological Sciences, Beijing, China; 4 Department of Botany, University of British Columbia, Vancouver, Canada; University of California-Davis, United States of America

## Abstract

Nucleocytoplasmic trafficking is emerging as an important aspect of plant immunity. The three related pathways affecting plant immunity include Nuclear Localization Signal (NLS)–mediated nuclear protein import, Nuclear Export Signal (NES)–dependent nuclear protein export, and mRNA export relying on MOS3, a nucleoporin belonging to the Nup107–160 complex. Here we report the characterization, identification, and detailed analysis of *Arabidopsis modifier of snc1, 11* (*mos11*). Mutations in *MOS11* can partially suppress the dwarfism and enhanced disease resistance phenotypes of *snc1*, which carries a gain-of-function mutation in a TIR-NB-LRR type *Resistance* gene. *MOS11* encodes a conserved eukaryotic protein with homology to the human RNA binding protein CIP29. Further functional analysis shows that MOS11 localizes to the nucleus and that the *mos11* mutants accumulate more poly(A) mRNAs in the nucleus, likely resulting from reduced mRNA export activity. Epistasis analysis between *mos3-1* and *mos11-1* revealed that MOS11 probably functions in the same mRNA export pathway as MOS3, in a partially overlapping fashion, before the mRNA molecules pass through the nuclear pores. Taken together, MOS11 is identified as a new protein contributing to the transfer of mature mRNA from the nucleus to the cytosol.

## Author Summary

In eukaryotic cells, mRNAs are synthesized in the nucleus where they are processed (capped, spliced, poly-adenylated, and carried toward the nuclear pore) prior to being exported to the cytoplasm for translation. Insights into mRNA export mechanisms have been gained mostly from studies on yeast and humans, while little is known about plant mRNA export. In a genetic screen for suppressors of the *Arabidopsis* autoimmune mutant *snc1*, we isolated and identified *mos11* (*modifier of snc1, 11*). *MOS11* encodes a protein with unknown function in the plant kingdom, and its closest relative in animals is the human RNA binding protein CIP29, whose function is unclear. We demonstrate that MOS11 is a nuclear protein and that *mos11* mutants accumulate more poly(A) mRNA in the nucleus, likely resulting from reduced mRNA export. Our data suggest that MOS11 functions before nuclear pore entry in the mRNA export pathway.

## Introduction

Plants utilize a two-layered immune system to recognize and combat pathogens. Conserved pathogen-associated molecular patterns (PAMPs), which are detected by plasma membrane localized plant pattern recognition receptors (PRRs), trigger a low amplitude defense response termed PAMP-triggered immunity (PTI) [1]. To suppress PTI, pathogens have evolved a suite of effectors (also called Avirulence or Avr proteins) [2]–[5]. The second layer of the plant immune response is initiated by the recognition of effectors or their effects, by cognate Resistance (R) proteins, leading to a strong defense response termed effector-triggered immunity (ETI) that culminates with hypersensitive response (HR) [1]: a programmed cell death event believed to restrict pathogen growth. Most cloned *R* genes encode Nucleotide Binding Leucine Rich-Repeat (NB-LRR) proteins with either a Toll/Interleukin1 receptor (TIR) or a Coiled-Coil (CC)-domain at their N terminus [6].

Mutant *snc1* (*suppressor of npr1-1, constitutive 1*) plants carry a gain-of-function mutation in a TIR-NB-LRR *R* gene. This mutation renders the snc1 protein auto-active without the presence of pathogens. As a consequence, *snc1* plants constitutively express *Pathogenesis Related* (*PR*) defense marker genes, accumulate high levels of defense phytohormone salicylic acid (SA), and are more resistant to the virulent bacterial pathogen *Pseudomonas syringae maculicola* (*P.s.m.*) ES4326 and the oomycete pathogen *Hyaloperonospora arabidopsidis* (*H.a*.) Noco2. Like most TIR-type R proteins, snc1 is fully dependent on EDS1 (Enhanced Disease Susceptibility 1) and PAD4 (PhytoAlexin Deficient 4) for its function [7], [8].

The *snc1* mutant results from a point mutation in the linker region between the NB and LRR domains. This gain-of-function mutation causes activation of the ETI pathways mediated by TIR-NB-LRR R proteins. The extreme dwarf morphology of *snc1* also makes it an ideal candidate to carry out genetic screens to investigate ETI components downstream of SNC1. Utilizing the unique autoimmune phenotypes of *snc1*, we conducted genetic screens to search for components contributing to R protein mediated immune responses. From the MOS (modifier of *snc1*) genetic screens, three nucleocytoplasmic pathways have been shown to affect plant immunity: Nuclear Localization Signal (NLS)-mediated nuclear protein import, Nuclear Export Signal (NES)-dependent nuclear protein export and mRNA export. MOS6, an importin α homolog involved in NLS-dependent protein import was shown to contribute to plant immunity [9]. More recently we identified MOS7, an integral nuclear pore component homologous to Nucleoporin88 (Nup88). Partial loss-of-function *mos7-1* mutant plants showed increased NES-dependent protein export and exhibited reduced nuclear accumulation of the important defense regulators EDS1, NPR1 (Non-expressor of Pathogenesis Related 1) and snc1 [10]. The contribution of mRNA export to plant immunity was demonstrated in the *mos3* mutant which exhibits defects in basal and R protein mediated immunity [10], [11]. MOS3/SAR3/AtNup96 is an integral nuclear pore component in the conserved Nup107–160 complex and was shown to be required for mRNA export [12].

Here we report the identification of *modifier of snc1, 11* (*mos11*), a T-DNA insertional mutant. Mutation in *MOS11* partially suppresses all the autoimmune phenotypes of *snc1*. *MOS11* encodes a protein with unknown function. However, it is evolutionarily conserved and shares homology with a human protein CIP29. Our data suggest that MOS11 localizes to the nucleus and functions in the same mRNA export pathway as MOS3.

## Results

### *mos11* partially suppresses the autoimmune phenotypes of *snc1*

**Figure 1:**
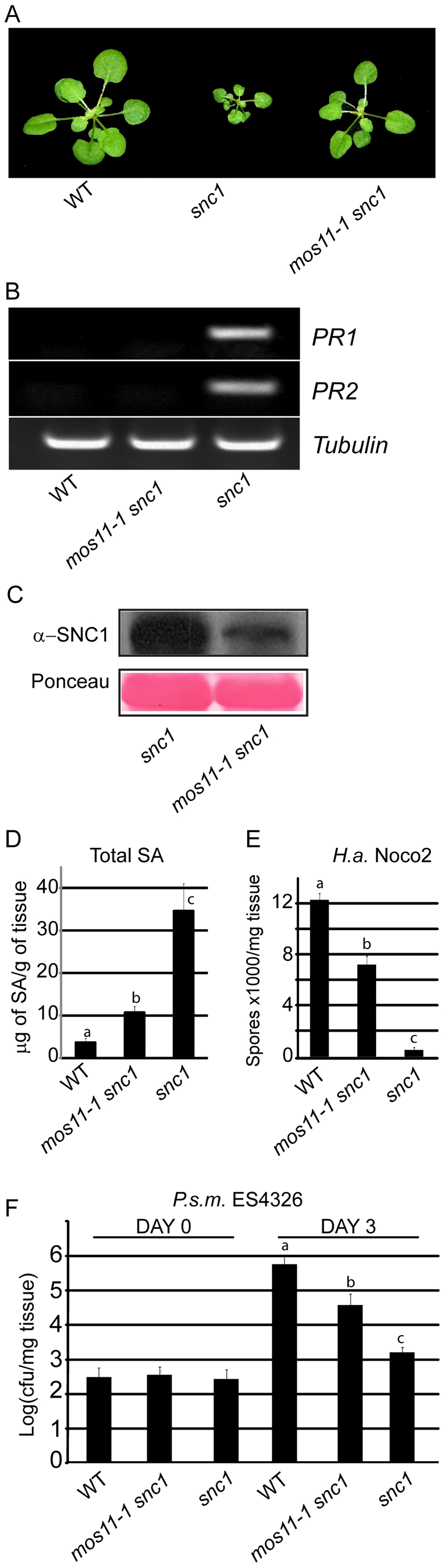
Suppression of *snc1*-associated dwarfism and enhanced resistance phenotypes by *mos11-1*. (A) Morphology of wild type (Col-0), *snc1* and *mos11-1 snc1*. The picture was taken with 4-week-old soil grown plants. (B) *PR1* and *PR2* gene expression as measured by RT-PCR in WT, *mos11-1 snc1* and *snc1* with *Tubulin* as loading control. RNA was extracted from 2-week-old plants grown on 1/2 MS medium and reverse transcribed to yield cDNA. *PR1*, *PR2*, and *Tubulin* were amplified by 27 cycles of PCR with equal amounts of total cDNA. PCR products were stained with ethidium bromide. (C) SNC1 protein level was evaluated by Western blot using an anti-SNC1 antibody [42]. Total protein was extracted from 4-week-old soil-grown plants. As a loading control, the Rubisco band is shown from a Ponceau stain. (D) Total SA was extracted from 4-week-old plants. The values presented are averages of three replicates with error bars showing standard deviations. Data were analyzed using one-way ANOVA. Different letters indicate statistically significant differences (*p*-value <0.0001). (E) Growth of *H.a.* Noco2 on WT, *mos11-1 snc1* and *snc1*. Two-week-old seedlings were sprayed with a conidiospore suspension of 5×10^4^ spores per ml of water. Spores were counted with a hemocytometer 7 days after infection. Data were analyzed using one-way ANOVA. Different letters indicate statistically significant differences (*p*-value <0.000001). (F) Growth of *P.s.m.* ES4326 in 5-week-old plants. Leaves were infiltrated with a bacterial suspension (OD_600_ = 0.0001). Leaf discs were collected immediately after infiltration 0 days post inoculation (DPI) and 3 DPI. Log-transformed values are averaged over four replicates with standard deviations (cfu = colony forming units). Data were analyzed using one-way ANOVA. Different letters indicate statistically significant differences (*p*-value <0.00001).

The *mos11-1* mutant was identified by T-DNA tagging in the *snc1* background [13]. Normally *snc1* plants are dwarf, have twisted dark green leaves, accumulate high levels of the defense phytohormone SA and are resistant to the virulent oomycete *H.a.* Noco2 and *P.s.m.* ES4326 bacteria. *mos11-1* partially suppressed the *snc1* morphology (Figure 1A). The *mos11-1 snc1* plants never grew to wild type size and their leaves remained curly (Figure 1A). In addition, semi-quantitative RT-PCR confirmed that endogenous *PR1* and *PR2*, defense markers acting downstream of *R* gene activation that are constitutively expressed in *snc1*, are greatly reduced in *mos11-1 snc1* (Figure 1B). In order to investigate how the *mos11* mutation suppresses *snc1* morphology and *PR* gene expression, we assessed whether the snc1 protein levels were affected in *mos11-1 snc1* plants. We observed a slight but consistent reduction in snc1 levels in *mos11-1 snc1* compared with *snc1* plants (Figure 1C). Total SA levels in *mos11-1 snc1* were drastically reduced compared with *snc1* plants, but were still significantly higher than in wild type plants (Figure 1D).

To assess whether the *mos11-1* mutation affects *snc1*-mediated resistance to virulent pathogens, we performed infection assays with the obligate biotrophic oomycete *H.a.* Noco2 and *P.s.m.* ES4326 bacteria. While *snc1* plants were resistant to *H.a.* Noco2 and showed very low sporulation, *mos11-1 snc1* displayed intermediate susceptibility to *H.a.* Noco2 (Figure 1E). *mos11-1 snc1* supported 13-fold more sporulation than *snc1*, however it was still significantly more resistant than the wild type (Figure 1E). This trend was also observed when we investigated susceptibility to *P.s.m.* ES4326 (Figure 1F). Taken together, these results show that *mos11-1* partially suppresses all the phenotypes of *snc1*.

### Identification of the molecular lesion in *mos11-1*

**Figure 2:**
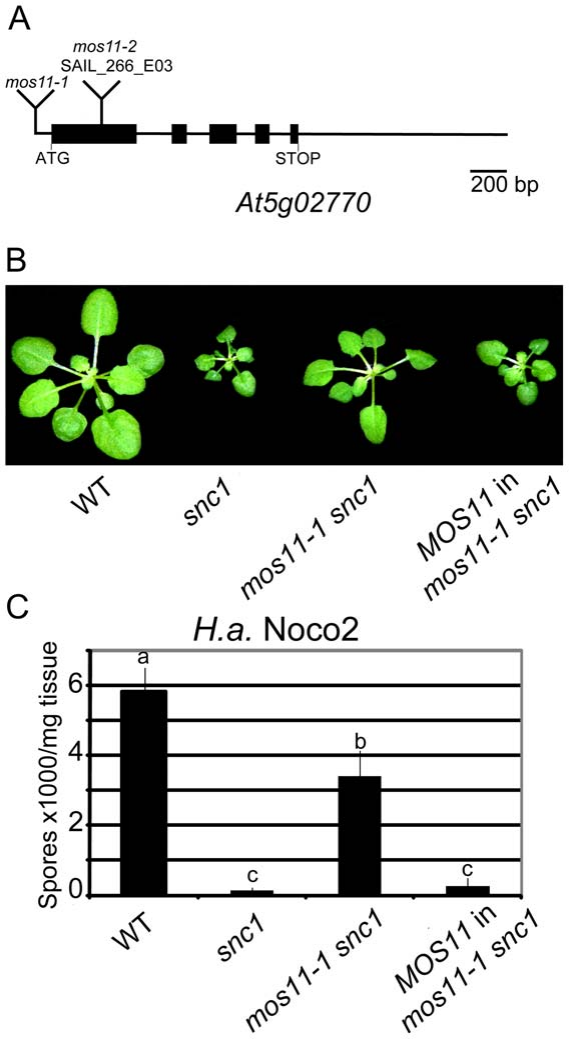
Complementation of *mos11-1 snc1* by *At5g02770*. (A) Gene structure of *At5g02770*. Exons are represented by black boxes and introns and UTRs are shown as solid lines. Locations of *mos11-1* and *mos11-2* (SAIL_266_E03) insertions are as indicated. (B) Morphology of WT, *snc1*, *mos11-1 snc1* and a representative transgenic line with *MOS11* (*At5g02770*) driven by its own promoter transformed into *mos11-1 snc1*. (C) Growth of *H.a.* Noco2 on WT, *snc1*, *mos11-1 snc1* and *MOS11* in *mos11-1 snc1*. The infection was carried out as in Figure 1E. Data were analyzed using one-way ANOVA. Different letters indicate statistically significant differences (*p*-value <0.001).

The T-DNA insertion causing the *mos11-1 snc1* phenotype was identified in the 5′ UTR of *At5g02770* using inverse PCR (Figure 2A). To confirm that the mutant phenotypes in *mos11-1 snc1* were caused by the T-DNA insertion, we cloned the wild type DNA of *At5g02770* with 1992 base pairs (bp) upstream of the start codon and transformed it into *mos11-1 snc1* plants. *At5g02770* fully complemented the *mos11-1 snc1* morphology (Figure 2B). When the T2 transgenic plants were challenged with *H.a.* Noco2, they were as resistant as *snc1* plants (Figure 2C). These data confirmed that *MOS11* is *At5g02770*.

Another T-DNA insertion allele (SAIL_266_E03) in the first exon of *At5g02770* was available from the Arabidopsis Stock Center (ABRC), which we named *mos11-2* (Figure 2A). The *mos11-1* single mutant was obtained from a backcross between *mos11-1 snc1* and the wild type Columbia ecotype (Col-0). The *mos11-1* and *mos11-2* single mutants had identical morphology but were slightly different than Col-0. When *mos11-1* was crossed with *mos11-2*, the two alleles failed to complement each other in all 29 F1 plants (data not shown), confirming that *mos11-2* and *mos11-1* carry lesions in the same gene. When *mos11-2* was crossed with *snc1*, the *mos11-2 snc1* double mutant exhibited a similar level of *snc1* suppression as *mos11-1* (data not shown), further supporting that *At5g02770* is *MOS11.*

### Subcellular localization of MOS11

*MOS11* is a single copy gene that encodes a 214 amino-acid protein with unknown function. Using BlastP, the human protein CIP29 (cytokine induced protein 29 kDa) was found to share 38% identity and 50% similarity over the entire length of the MOS11 protein. It is striking that islands of positively charged residues, arginine and lysine, are highly conserved (see alignment in Figure S1). Although MOS11 does not have predicted NLS (Nuclear Localization Signal) motifs, it is predicted by WoLF PSORT (http://wolfpsort.org/) to be a nuclear protein.

**Figure 3:**
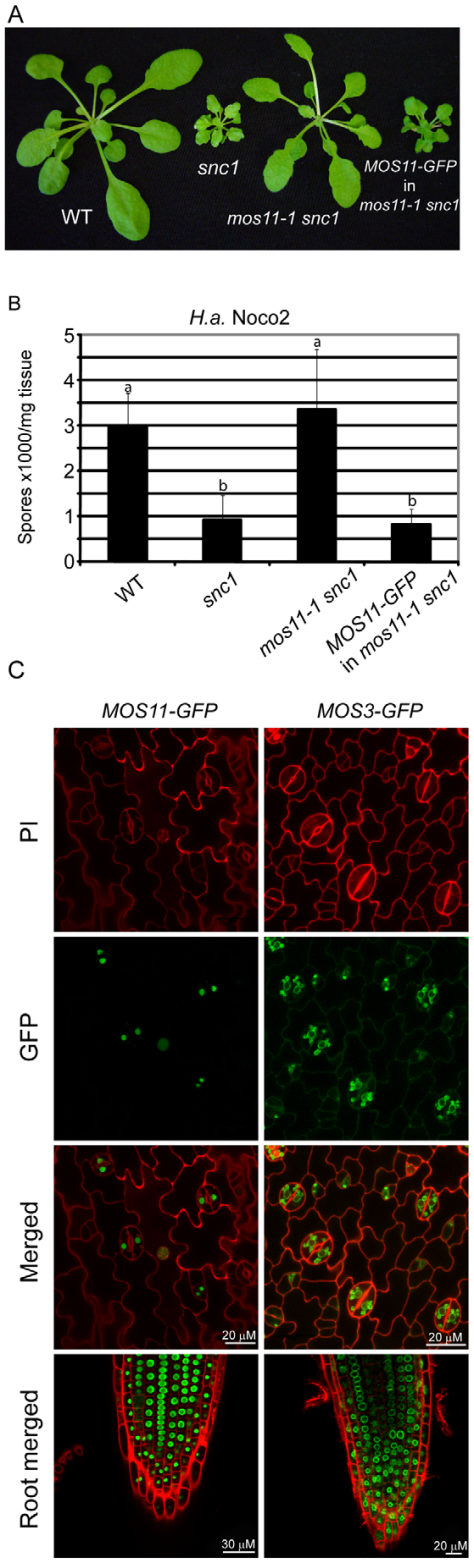
MOS11-GFP is localized to the nucleus. (A) Morphology of WT, *snc1, mos11-1 snc1* and a transgenic line with *MOS11-GFP* driven by its own promoter transformed into *mos11-1 snc1.* (B) Growth of *H.a.* Noco2 on WT, *snc1*, *mos11-1 snc1* and *MOS11-GFP* in *mos11-1 snc1.* The infection was carried out as in Figure 1E. Data were analyzed using one-way ANOVA. Different letters indicate statistically significant differences (*p*-value <0.00001). (C) MOS11-GFP and MOS3-GFP fluorescence as observed by confocal microscopy in leaf cells and root cells. Cell walls were stained with propidium iodine (PI).

To determine the biochemical function of MOS11, we first investigated its subcellular localization. When *MOS11-GFP* with its native promoter was transformed into *mos11-1 snc1*, among the 9 T1 progeny, all showed *snc1*-like morphology (Figure 3A). The T2 transgenic plants also displayed *snc1*-like enhanced resistance to *H.a.* Noco2 (Figure 3B). These data indicate that *MOS11-GFP* largely reflects endogenous MOS11 in the transgenic line, and therefore, the subcellular localization of MOS11-GFP should be identical to MOS11. As shown in Figure 3C, in contrast to the MOS3-GFP control, which localizes to the nuclear envelope, strong MOS11-GFP signal was observed in the nuclei of leaf epidermal and root cells, however it appears to be excluded from the nucleolus. Nuclear localization of the MOS11-GFP signal was observed in guard cells as well. Thus we conclude that MOS11 is a nuclear protein.

### MOS11 is required for mRNA export

**Figure 4:**
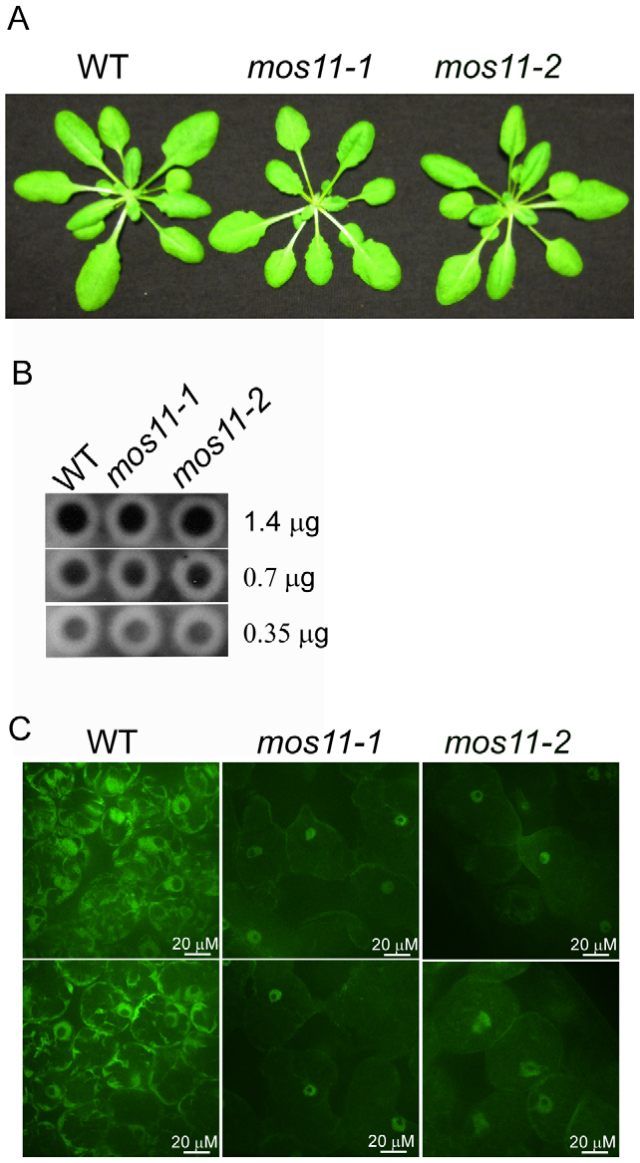
mRNA export is impaired in *mos11* plants. (A) Morphology of 4-week-old soil-grown WT, *mos11-1* and *mos11-2* plants. (B) Dot blot of WT, *mos11-1* and *mos11-2*. The same amount of WT, *mos11-1*, and *mos11-2* total RNA were applied to the Hybond-N+ membrane and hybridized by ^32^P-ATP labeled 18-mer oligo dT. The amount of total RNA loaded to each dot was indicated on the right of the blot. The radioactive signal was detected by a phosphor-imager. (C) Whole mount *in situ* mRNA localization of 7-day-old seedlings probed with 48-mer oligo d(T) labeled with Alexa 488. Observations were done at 63× magnification and microscope settings were identical for all samples.

**Figure 5:**
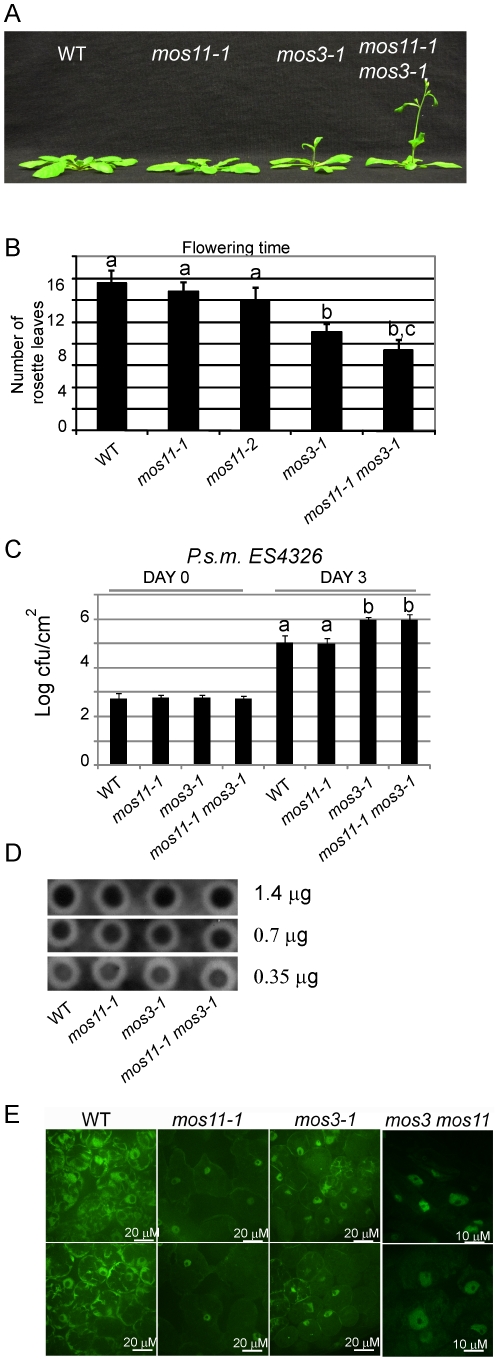
*mos11-1 mos3-1* double mutant is impaired in flowering time, basal defense, and mRNA export. (A) Morphology of 4-week-old soil-grown WT, *mos11-1, mos3-1* and *mos11-1 mos3-1* plants. (B) Quantitative flowering time analysis assessed by counting the number of rosette leaves on 4-week-old soil-grown plants. (C) Growth of *P.s.m.* ES4326 in 5-week-old plants. The infection was carried out as in Figure 1F. Data were analyzed using one-way ANOVA. Different letters indicate statistically significant differences (*p*-value <0.00001). (D) Dot blot of WT, *mos11-1*, *mos3-1* and *mos3-1 mos11-1*. The same amount of WT, *mos11-1*, *mos3-1* and *mos3-1 mos11-1* total RNA were applied to the Hybond-N+ membrane and hybridized by ^32^P-ATP labeled 18-mer oligo dT. The amount of total RNA loaded to each dot is indicated to the right of the blot. The radioactive signal was detected by the phosphor-imager. (E) Whole mount *in situ* mRNA localization of 7-day-old seedlings probed with oligo d(T) labeled with Alexa 488. Observations were done at 63× magnification and microscope settings were identical for all samples.

In humans, CIP29 was shown to bind RNA (23). The two known interacting protein partners of CIP29 are the RNA helicase DDX39 and FUS/TLS. DDX39 shows 91% identity with UAP56, a *Drosophila* RNA helicase involved in mRNA export [14]. DDX39 also interacts with ALY and FUS/TLS, which were both shown to contribute to mRNA export [15]–[17]. These associations prompted us to investigate whether MOS11 also contributes to mRNA export. We first used dot blot hybridization to check whether the total mRNA level was affected in *mos11-1* and *mos11-2* plants. *mos11-1* and *mos11-2* are morphologically indistinguishable (Figure 4A). Decreasing RNA concentrations were used to avoid saturation. No visible differences in total RNA levels were observed (Figure 4B). To assess whether mRNA export from the nucleus to the cytoplasm was affected in *mos11-1* and *mos11-2* plants, we carried out poly(A) *in situ* hybridization with a 48-mer oligo d(T) 5′-end labeled with Alexa 488. Poly(A) signals were observed in the nucleus of Col-0 and the cytosol of all labeled cells (Figure 4C). When *mos11-1* and *mos11-2* were analyzed, most poly(A) signal was found in the nucleus, indicating that mRNA export is diminished in *mos11* plants, resulting in mRNA accumulation in the nucleus (Figure 4C and Figure S2). Thus MOS11 is likely a contributing factor in the mRNA export pathway. Note that nucleolar oligo d(T) signal is weak in all *in situ* experiments (Figure 4C and Figure 5E), a region where MOS11-GFP was also absent (Figure 3C).

### MOS11 functions in the same mRNA export pathway as MOS3 in a partially overlapping manner

Since *mos3* was previously shown to affect mRNA export [12], we investigated the relationship between *mos3-1* and *mos11-1* using epistasis analysis. The morphological phenotypes associated with both single mutants are quite different; *mos3-1* shows long and narrow leaves and flowers early, whereas *mos11-1* has mostly wild type-like morphology. The *mos3-1 mos11-1* double mutant displayed a *mos3*-like morphology with leaves that are slightly narrower and longer than those of the *mos3* single mutant, but flowered slightly earlier than *mos3-1* or *mos11-1* (Figure 5A and 5B). These data suggest that in regard to flowering time regulation, MOS3 and MOS11 seem to play partially overlapping roles.

In order to assess the relationship between *mos3* and *mos11* in immunity, we evaluated the response of *mos3-1*, *mos11-1* and *mos11-1 mos3-1* to the virulent bacteria *P.s.m.* ES4326. Virulent pathogens, ones not carrying *Avirulence* genes recognized by the plant R proteins, were used to evaluate the contribution of basal resistance to immunity. Both *mos3-1* and *mos11-1 mos3-1* plants exhibited enhanced susceptibility when infiltrated with virulent *P.s.m.* ES4326 and supported higher bacterial growth, whereas the response of the single *mos11-1* mutant was similar to that of the wild type (Figure 5C), suggesting that *MOS11* is not involved in basal resistance. No further enhanced susceptibility was observed for *mos11-1 mos3-1*, indicating that these two genes function in the same pathway to regulate immunity. It should be noted that due to the quantitative limit of infection assays, possible minor additive effects like those observed for the flowering time phenotype might have been missed.

To test whether *mos11* affects R protein mediated resistance, we inoculated Col-0, *mos11-1* and *mos11-2* with *Pseudomonas syringae* pv *tomato* (*P.s.t.*) DC3000 strains expressing *Avirulence* genes *avrRps4*, *avrRpm1*, *avrRpt2* or *avrPphB*. Avirulent pathogens carry *Avirulence* genes whose products or the effects of the products in the plant cells are recognized by plant R proteins. These pathogens are used to evaluate the integrity of ETI in plants. Bacterial growth was not affected in the single mutants for any of the R protein mediated pathways (Figure S3). Thus we conclude that MOS11 is not involved in the ETI response mediated by these R proteins.

**Figure 6:**
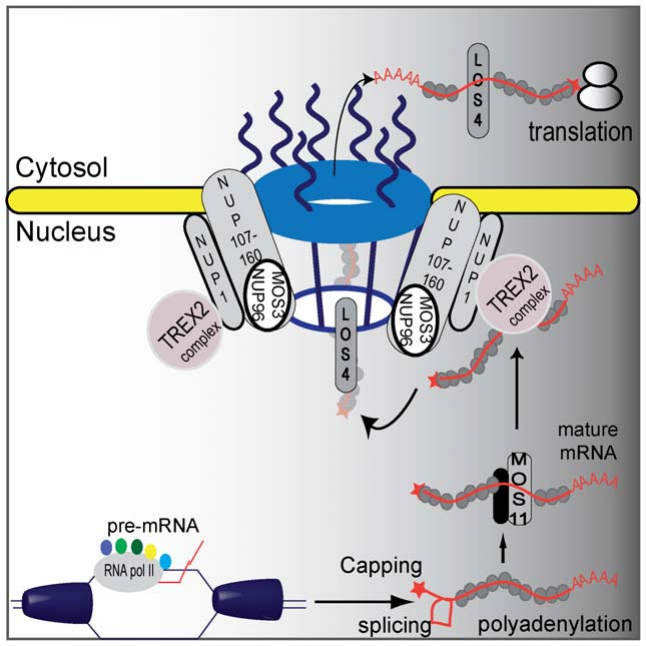
A proposed plant mRNA export pathway. DNA helicase unwinds DNA and RNA polymerase II and co-activators proceed with transcription and mRNA maturation. Pre-mRNA is capped, polyadenylated, and spliced. Mature mRNA is then carried by MOS11 and its partners to TREX2, which associates with NUP1. LOS4 then binds the mRNA and passes the mature mRNA through the nuclear pores with the help of the NUP107–160 complex to the cytosol where it can be translated.

We then analyzed the mRNA export status in the *mos11-1 mos3-1* double mutant compared with the single mutants. Similar to the *mos3-1* and *mos11-1* plants, the poly(A) signal in the double mutant was mostly observed in the nucleus. However, further poly(A) accumulation was not detected in the nuclei of the double mutant (Figure 5E and Figure S2). It should be noted that in all the mRNA export deficient mutants tested, some signal, albeit much weaker than the wild type, was always observed in the cytosol, which explains why these mutations are not lethal and the plants can successfully complete their life cycle. Together, these data suggest that MOS3 and MOS11 function in the same mRNA export pathway, possibly in a partially overlapping manner. Since MOS3 is part of the nuclear pore while MOS11 localizes inside the nucleus, MOS3 probably functions downstream of MOS11 in mRNA export (Figure 6).

## Discussion

To search for components in R protein mediated immunity, we performed genetic screens for suppressors of the autoimmune mutant *snc1*. *mos11-1* was identified from a T-DNA mutagenized *snc1* population. Mutation in *MOS11* partially abolishes the dwarf stature, elevated SA levels, and enhanced resistance to *H.a.* Noco2 and *P.s.m.* ES4326 of *snc1*. *MOS11* encodes a protein with unknown function in the plant kingdom, however, it shares 50% similarity with human CIP29. Similarities and identities are more striking in islands of conserved positively charged residues; namely lysine and arginine (Figure S1). These basic islands are known to be present in nucleic acid binding proteins directly involved in DNA/RNA binding [18]–[20].

There are a limited number of reports on human CIP29, which was initially named HCC-1 after being isolated from the hemofiltrate of patients with chronic renal failure [21] and later renamed CCL14a [22]. Overexpression of CIP29 in human embryonic kidney cell line results in slower growth. Biochemical characterization demonstrated that CIP29 could bind RNA on its own [23]. In yeast two-hybrid assays, CIP29 interacted with two DEAD-box RNA helicases, BAT1 and DDX39 [24].

Hints on the potential biological functions of CIP29 came from the study by Sugiura et al. (2007). They showed that the ATP-dependent DEAD-box RNA helicases DDX39 can co-immunoprecipitate CIP29, confirming the earlier yeast two-hybrid interaction reported by Leaw et al. (2004). Additionally they demonstrated that CIP29 can bind RNA on its own [23], and more importantly, it enhances the RNA unwinding activity of DDX39. The same group also identified FUS/TLS, a nucleic acid binding protein participating in both transcription and splicing as a physical interactor of CIP29 [23]. In addition, they detected ALY, a well-characterized mRNA export factor, in the DDX39 immunoprecipitate. CIP29 localized mainly to the nucleus although small amounts were also detected in the cytosol [25]. Very recently, Dufu et al. (2010) reported that CIP29 physically associates with ALY via UAP56 in an ATP-dependent manner to form a trimeric complex. In addition they demonstrated that efficient recruitment of CIP29 to the mRNA is splicing- and cap-dependent [26]. The functions of the CIP29 interactors, its affinity for RNA and the conserved island of positively charged residues suggest a potential role of CIP29 in RNA processing and/or RNA export, although mRNA export defects have not been shown for a *cip29* mutant. Through knockout analysis with *mos11* mutants, we demonstrate that MOS11 is indeed required for mRNA export. We speculate that its human counterpart CIP29 probably has a similar function.

In eukaryotic cells, transcription and translation take place in two separate compartments, which allows fine-tuned regulation of biological processes. For proteins to be translated in the cytosol, the mRNA molecules must first be properly processed, assembled into an export competent mRNA ribonucleoprotein (mRNP) particle and transferred through the nuclear pore. This assembly starts at the onset of transcription. Several proteins bind to the nascent pre-mRNA. These proteins can be involved in transcription [27], capping, splicing [28], polyadenylation [29] or binding to nuclear pore proteins [30]. Most of the knowledge on mRNA export has been gained from studies of human and yeast cells. Little is known about the mechanisms of plant mRNA export (for reviews see [31], [32]), although it is believed to share high similarity with yeast and human pathways.

In plants, several nuclear pore complex mutants have been shown to exhibit defects in mRNA export [12], [31]–[37]. In *Arabidopsis*, previously reported components shown to participate in mRNA export all localize to the nuclear envelope; these include MOS3/AtNup96, AtNup160, LOS4, AtNup1 and AtTHP1. *LOS4* encodes a DEAD-box RNA helicase enriched at the nuclear rim and it is also present in the cytosol. Two point mutation alleles of *los4/cryophyte* were shown to affect mRNA export [38]. More recently orthologs of the TREX-2 complex, which is necessary for mRNA export in yeast and humans, have been identified in *Arabidopsis*. The TREX-2 complex, which is tethered to the nuclear pore complex via Nup1 in yeast, is composed of Thp1, Sac3, Cdc31 and Sus1. AtNUP1 and AtTHP1 were shown to be necessary for mRNA export, but the other three proteins do not appear to be involved in mRNA export despite evidence of a physical interaction with other TREX-2 subunits [36]. Another protein, TEX1, which is not part of the THO/TREX core complex but part of a complex associated with the TREX complex is also found in *Arabidopsis* [37]. In *Drosophila* and human, TEX associates with the helicase UAP56 and the mRNA export factor ALY [39], [40]. However, co-IP experiments using HA-tagged AtTEX did not pull-down ALY or UAP56 in *Arabidopsis*, but they did retrieve THO1, THO2, THO5, THO6 and THO7 [37].

Subcellular localization of MOS11-GFP revealed that MOS11 is a nuclear protein. In *mos11-1*, *mos11-2* and *mos3-1*, mRNA export was similarly affected. Epistasis analysis between *mos11-1* and *mos3-1* suggested that these two genes function in the same mRNA export pathway. Thus we drew a simplified model of the mRNA export pathway in plants (Figure 6) where MOS11 likely functions in the early steps of the mRNA export before mRNA exits the nucleus through the nuclear pore. This is consistent with their respective subcellular localization where MOS11 is nuclear and MOS3 is limited to the nuclear rim. This indicates that MOS11 has access to the mRNA prior to MOS3. Since MOS11 is conserved in eukaryotes, its human homolog CIP29 probably also functions in a similar step in mRNA export as suggested by Dufu et al. [26].

The reason why *mos11* suppresses *snc1* phenotypes is not clear. *mos11* single mutant plants, although slightly different from the wild type, grow to full stature, produce seeds and complete their life cycle successfully. This shows that the observed impairments in mRNA export in *mos11* are not sufficient to significantly affect ontology. The *snc1* mutation involves an extensive transcriptional reprogramming that results in the dramatic phenotypes displayed by *snc1* plants. Among the changes observed following the auto-activation of *snc1* are the induction of defence gene markers and a strong stimulation of the SA biosynthetic pathway. These changes are associated with dwarf stature and increased resistance to pathogens. It is conceivable that MOS11 could promote mRNA export of genes that are up-regulated following defence activation, possibly including *R* genes. In support of this hypothesis, dot blot analysis demonstrated that *mos11-1* does not affect the overall level of transcription since all lines analyzed had similar levels of total mRNA. However *mos11-1* does have an effect on snc1 protein level in *mos11-1 snc1*, possibly resulting from impaired *SNC1* mRNA export leading to less mRNA for translation of the protein (Figure 1C).

The detailed mechanism of MOS11 in mRNA export is not clear. Since CIP29 was recently shown to interact with ALY and UAP56 in an ATP-dependent manner [26], and it was also shown to enhance the RNA unwinding activity of DDX39 [23], we speculate that the function of MOS11 is likely to act as a co-chaperone to plant homologs of UAP56 and ALY to co-catalyze mRNA unwinding prior to its nuclear export. The specific targets of MOS11 await further investigation.

## Materials and Methods

### Plant growth, SA measurement, RT-PCR analysis, pathogen infections, and T-DNA screens

All plants were grown under a 16 h light/8 h dark regime. SA extractions and measurements were carried out as previously described [41]. RNA was extracted from 2-week-old plate-grown seedlings on half Murashige and Skoog (½ MS) media using the Totally RNA kit (Ambion). 200 ng of RNA was used for reverse transcription using Superscript II (Invitrogen). RT-PCR analysis of *PR1* and *PR2* expression was carried out as previously described [8]. Tubulin was used as an uninduced loading control with primers 5′ACGTATCGATGTCTATTTCAACG3′ and 5′ATATCGTAGAGAGCCTCATTGTCC3′. Pathogen assays with *H.a.* Noco2 and *P.s.m.* ES4326 were carried out as previously described [7]. T-DNA screen in *snc1* was described previously [13].

### Total protein extraction and Western blot

Total protein was extracted from 4-week-old soil-grown plants using the extraction buffer containing 100 mM Tris-HCl (pH 8), 0.2% SDS and 2% β-mercaptoethanol. 30 µl of total protein was separated on 8% SDS-PAGE and transferred onto nitrocellulose membrane. For SNC1 total protein level, the membrane was probed with purified anti-SNC1 antibody, which was generated against a SNC1-specific peptide in rabbit [42].

### MOS11-GFP subcellular localization

The full-length genomic *MOS11* gene plus 1992 bp upstream of its start codon was cloned in frame to the N-terminus of GFP in a modified pCambia1305 vector in which the *GUS* gene had been replaced by *GFP*. Plant transformation was carried out by *Agrobacterium in planta* dipping [43] and transformants were selected on ½ MS supplemented with 30 µg/ml hygromycin. T1 and T2 transgenic plants were then observed with a confocal microscope with propidium iodine as a cell wall/dying nuclei marker.

### Dot blot hybridization

The total RNA was extracted by RNAiso Plus (Takara, Cat No. D9108A). The RNA samples were quantified by spectrophotometry and confirmed by agarose gel electrophoresis. The RNA samples were prepared and applied to the Hybond-N+ membrane (Amersham Cat. No. RPN303B). The membrane was cross-linked for 2 minutes at 70000 micro-joules/cm^2^ and baked for 2 hours at 80°C. The membrane was pre-hybridized for 1 hour at 37°C in Hyb-50 (Mylab Corporation, Beijing, P. R. C.). The 18-mer oligo-dT was labeled with ^32^P by the T4 poly-nucleotide kinase (NEB, Cat. No. M0201) and added to the pre-hybridization buffer. After 16 hours of hybridization, the membrane was washed following Amersham Hybond-N+ manual. The signals were detected by phospho-imager using a Typhoon scanner.

### Whole mount *in situ* total mRNA localization

We adopted a protocol similar to that described in Parry et al. (2006). Briefly, 4–7 day-old plate-grown seedlings were immersed in 1 ml fixation cocktail (50% fixation buffer consisting of 120 mM NaCl, 7 mM Na_2_HPO_4_, 3 mM NaH_2_PO_4_, 2.7 mM KCl, 0.1% Tween 20, 80 mM EGTA, 5% formaldehyde, 10% DMSO and 50% heptane) in a glass container and gently agitated for 30 minutes at room temperature. The sample was dehydrated twice for 5 minutes each in 100% methanol, three times for 5 minutes each in 100% ethanol and incubated for 30 minutes in ethanol:xylene (50∶50) with gentle agitation. The samples were washed twice in ethanol for 5 minutes each and twice in methanol for 5 minutes each before the samples were incubated 5 minutes in methanol: fixation buffer without formaldehyde (50∶50) and fixed in fixation buffer with 5% formaldehyde for 30 minutes. Samples were rinsed twice for 5 minutes each with fixation buffer without formaldehyde and incubated for 5 minutes with PerfectHyb Plus (Sigma). 1 ml of new hybridization buffer was added and pre-hybridized at 50°C for 1 hour. Finally, 5 pmol of 5′ end-labeled Alexa-488 48-mer oligo d(T) (Invitrogen) was added and incubated overnight in the dark. The final samples were mounted in water and fluorescence was observed with a spinning disc microscope at a magnification of 63X using the following settings: Volocity 5.1 software, Hamamatsu C9100-50 camera, the exposure time was set to 100 ms and the sensitivity was adjusted to 176. Settings were the same for all samples.

## Supporting Information

Figure S1 Amino acid sequence alignment of MOS11 and its homologs in human, mice, rice, corn, poplar, and grape vine. Identical amino acids are shaded dark and similar amino acids are shaded light. Asterisks indicate highly conserved positively charged residues.
(1.38 MB TIF)

Figure S2 Quantification of the amount of fluorescent signal in the nucleus of the genotypes described in Figure 4C and Figure 5E, reflecting the relative amount of mRNAs.
(0.11 MB TIF)

Figure S3 Growth of *P.s.t.* DC3000 carrying respective Avr effectors in 5-week-old plants. The infection was carried out as in Figure 1F. Data were analyzed using one-way ANOVA. Different letters indicate statistically significant differences (*p*-value <0.00001).
(0.26 MB TIF)
