# Supplementary figures and images for: MOS11: A New Component in the mRNA Export Pathway
Source: PLoS Genet. 2010 Dec 23;6(12):e1001250. doi: 10.1371/journal.pgen.1001250 (PMC3009657; doi:10.1371/journal.pgen.1001250)

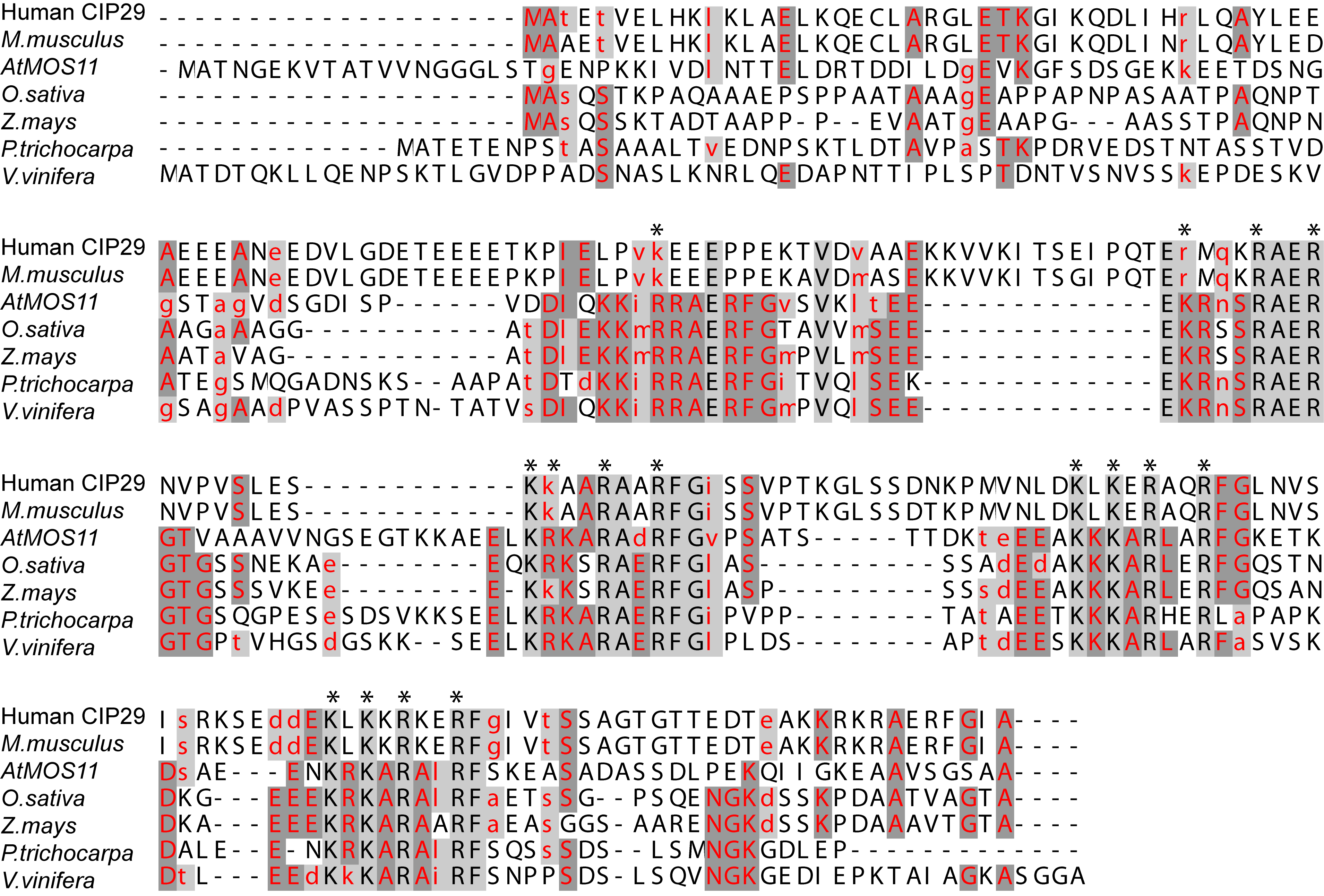

Supplement: Figure S1 — Amino acid sequence alignment of MOS11 and its homologs in human, mice, rice, corn, poplar, and grape vine. Identical amino acids are shaded dark and similar amino acids are shaded light. Asterisks indicate highly conserved positively charged residues. (1.38 MB TIF) [file pgen.1001250.s001.tif]

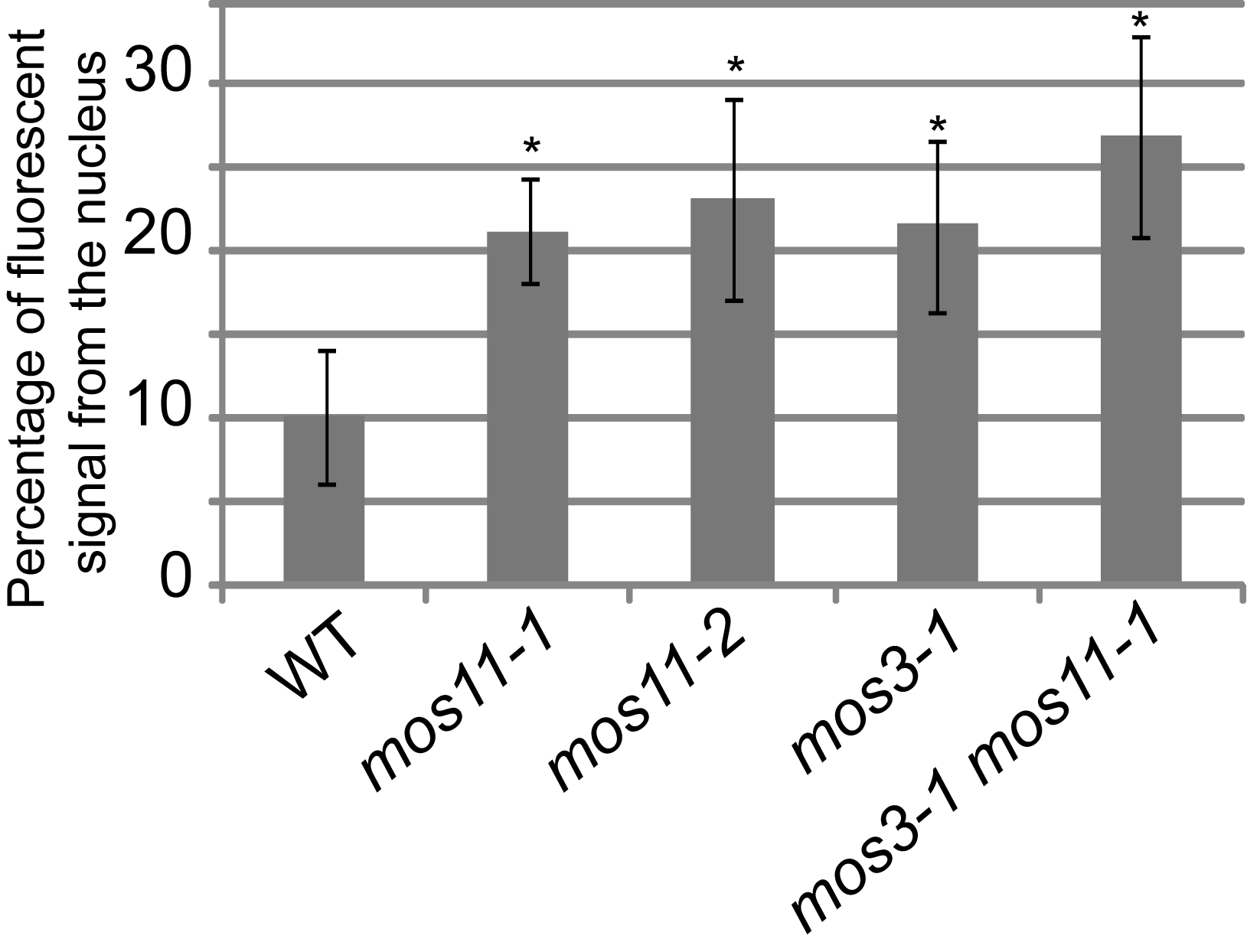

Supplement: Figure S2 — Quantification of the amount of fluorescent signal in the nucleus of the genotypes described in Figure 4C and Figure 5E, reflecting the relative amount of mRNAs. (0.11 MB TIF) [file pgen.1001250.s002.tif]

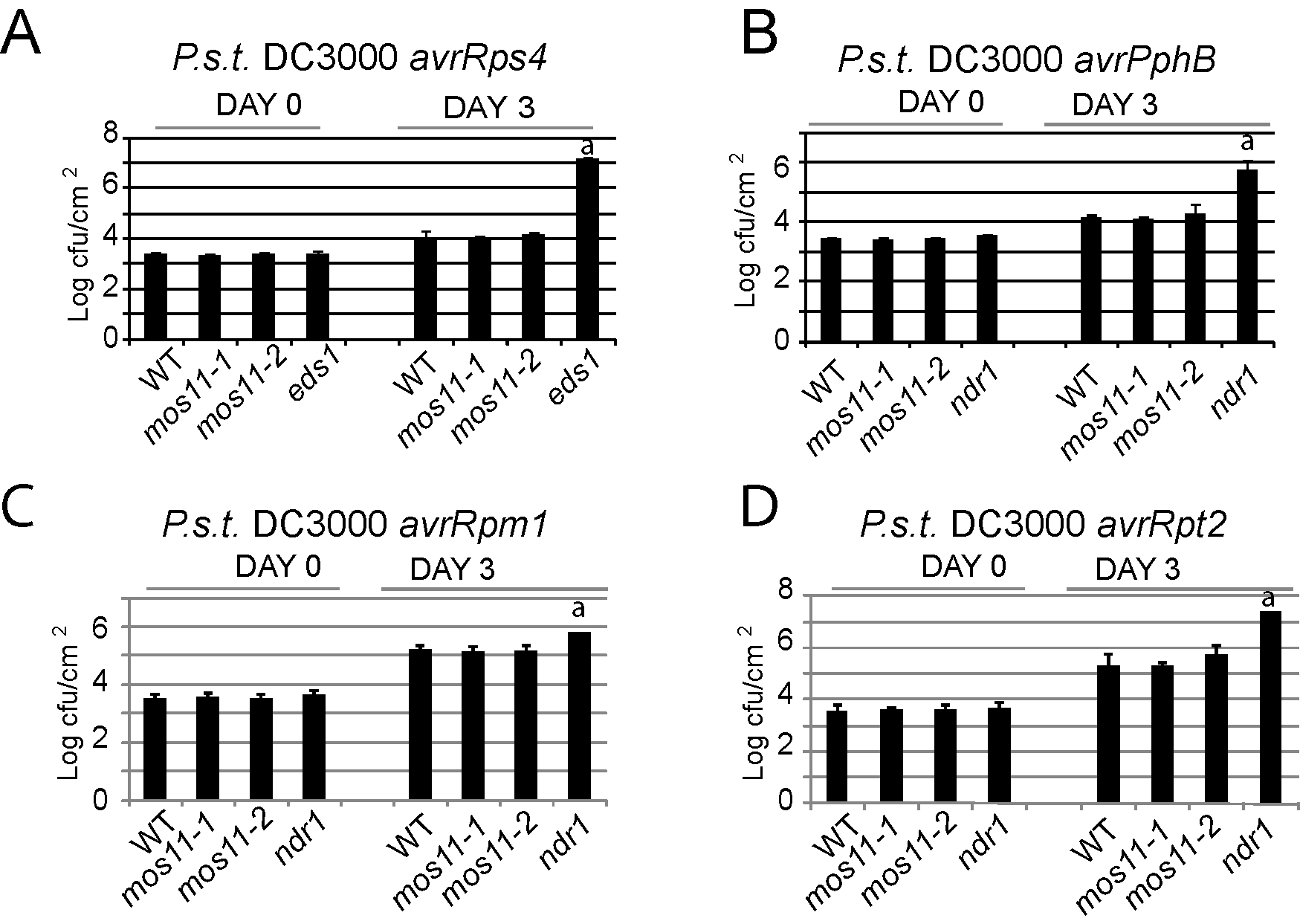

Supplement: Figure S3 — Growth of P.s.t. DC3000 carrying respective Avr effectors in 5-week-old plants. The infection was carried out as in Figure 1F. Data were analyzed using one-way ANOVA. Different letters indicate statistically significant differences (p-value <0.00001). (0.26 MB TIF) [file pgen.1001250.s003.tif]
